# Multifactorial mechanisms of the pathogenesis of methicillin-resistant *Staphylococcus hominis* isolated from bloodstream infections

**DOI:** 10.1007/s10482-017-1007-3

**Published:** 2017-12-20

**Authors:** Ewa Szczuka, Sylwia Krzymińska, Natalia Bogucka, Adam Kaznowski

**Affiliations:** Department of Microbiology, Faculty of Biology, Institute of Experimental Biology, Adam Mickiewicz University in Poznań, ul. Umultowska 89, 61-614 Poznań, Poland

**Keywords:** *Staphylococcus hominis*, Antibiotic resistance genes, Adhesion, Invasion, Cytotoxicity

## Abstract

*Staphylococcus hominis* is a species of the coagulase-negative staphylococci. It has been designated as a potential pathogen but so far the pathogenic mechanisms of this bacterium have not been determined. We studied 30 clinical isolates of methicillin-resistant *S. hominis*, which were previously examined for biofilm forming properties. The results of this study revealed that all these *S. hominis* strains had the ability to adhere to HeLa cells. Over 40% of the *S. hominis* strains invaded epithelial cells. The invasion index ranged from 0 to 41.5%. All isolates exhibited the cytotoxic activity of extracellular factors, which caused the destruction of epithelial cells. More than 90% of these methicillin-resistant strains contained at least one aminoglycosides resistance gene. The *ant(4′)*-*I* gene was found in 63% of the isolates, *aac(6′)/aph(2″)* in 20% and *aph(3′)*-*IIIa* in 47%. Two strains were assigned to SCC*mec* type VIII and three to SCC*mec* type III. The remaining isolates (83%) harboured a non-typeable SCC*mec* type. The *mec* complex A was predominant in this species. The results indicate that the pathogenicity of *S. hominis* may be multifactorial, involving adhesion, invasion and the activity of extracellular toxins, which cause damage to the host epithelium.

## Introduction

*Staphylococcus hominis* is a species of the Coagulase-negative staphylococci (CoNS). Among CoNS, *S. hominis* is one of the three most frequently identified isolates recoverable from the blood of hospitalised patients (Spanu et al. 2003; Chaves et al. 2005; Becker et al. 2014). These bacteria are recognised as potentially opportunistic pathogens and may cause bloodstream infections, endocarditis, peritonitis, osteomyelitis, bone and joint infections (d’Azevedo et al. 2008; Sorlozano et al. 2010; Ruiz de Gopegui et al. 2011; Becker et al. 2014). The majority of the infections caused by staphylococci are associated with the use of indwelling medical devices (Rodhe et al. 2006; Sorlozano et al. 2010; Mendoza-Olazarán et al. 2013). The exact mechanisms of *S. hominis* pathogenicity have still not been determined.

The rise of drug-resistant strains is a serious problem in the treatment of infections caused by *S. hominis* (Sorlozano et al. 2010; Ruiz de Gopegui et al. 2011; Szczuka et al. 2016a, b). Methicillin-resistance is associated with the presence of the *mecA* gene, which is located on a mobile genetic element, called the staphylococcal cassette chromosome (SCC*mec*). The *mecA* gene encodes a penicillin-binding protein (PBP2a). As a result of *mecA* expression, beta-lactam antibiotics are not effective against MRSA. To date, eleven SCC*mec* types have been described, based on the class of the *mec* gene complex (A–E) and the *ccr* gene complex (IWG 2009). The *ccr* genes encoding recombinases responsible for the integration and excision of SCC*mec* from the chromosome. According to the literature, SCC*mec* elements, in CoNS, are highly diverse in their genetic content. The SCC*mec* can harbour multiple *ccr* allotypes and/or the untypeable *ccr* allotype, and a new combination of the *mec* complex and the *ccr* complex (IWG 2009; Ruppé et al. 2009; Mendoza-Olazarán et al. 2013; Shore and Coleman 2013; Bouchami et al. 2012; Szczuka et al. 2016a, b). Methicillin-resistant staphylococci are most often resistant to a number of widely used antimicrobial agents. For this reason, vancomycin is usually the antibiotic of choice in the treatment of staphylococcal infections. When combination therapy is required, aminoglycosides are used, because of their synergistic bactericidal effect in combination with vancomycin. Resistance to aminoglycosides is usually due to drug inactivation by cellular aminoglycosides-modifying enzymes (AMEs). The bifunctional enzyme AAC(6′)/APH(2″) encoded by the gene *aac*(6′)-Ie-*aph*(2″)-Ia and located on transposon *Tn4001,* modifies all clinically available aminoglycosides, except streptomycin (Ramirez and Tolmasky 2010). The APH(3′)-III enzyme encoded by the *aph(3′)*-*IIIa* gene mediates resistance to kanamycin, neomycin and amikacin. The ANT(4′)-I enzyme encoded by the *ant(4′)*-*Ia* gene and occurring on plasmid pUB110, inactivates tobramycin, kanamycin, neomycin and amikacin (Ramirez and Tolmasky 2010; Wendlandt et al. 2013).

In this study, we analysed mechanisms of *S. hominis* virulence. Moreover, the frequency of antibiotic resistance genes and the diversity of the *SCCmec* types were also determined.

## Materials and methods

### Bacterial strains

Thirty strains isolated from the blood of hospitalised patients were regarded as the causative agents of blood stream-infections (at least two blood cultures taken from separate vein puncture were positive). Patients were treated in the Intensive Care Unit, Neonatal Unit and Orthopedic Ward. Strains were identified using a VITEK 2 system (bioMérieux, France). Isolates were stored at − 70 °C. Previously, these strains were tested for biofilm forming properties (Szczuka et al. 2015). Resistance to methicillin was determined by a cefoxitin disc test.

### Epithelial cell line

Human epithelial cells from cervix (HeLa cells) were kindly provided from Department of Molecular Virology from Adam Mickiewicz University in Poznań, Poland. The cells were cultivated in RPMI with 5% fetal bovine serum (FBS, Gibco) and 2 mM glutamine (Difco), 50 Iu/ml penicillin, 100 µg/ml streptomycin (Nawrot et al. 2010).

### Bacterial adhesion and invasion

Adhesion and invasion ability were performed in a quantitative assay using the gentamicin/lysostaphin protection method, with modifications (Krzymińska et al. 2012a, b, 2015). For the analysis of bacterial adhesion, the infected HeLa cells were lysed using 0.1% Triton X-100. The total of adherent and invasive bacteria was determined by plating serial dilutions of the lysates on BHI agar. Adherence was expressed as an index, which was designated as a number of colony factor units (CFU) of the associated bacteria, per 1 ml (1 × 10^6^) cells. For the assessment of the bacterial invasion of HeLa cells, the infected cells were incubated for 2 h with RPMI, with 300 μg/ml of gentamicin (Krka) and 100 μg/ml of lysostaphin (Sigma) to kill extracellular bacteria. Lysostaphin is unable to enter eukaryotic cells and also gentamicin does not penetrate eukaryotic cells and is effective in eradicating extracellular bacteria, even those with aminoglycoside resistance. After three washes with PBS, the cells were lysed with Triton X-100 and bacterial counts were determined by plating serial dilutions of the lysates on BHI agar. Bacterial invasion was expressed as an index that represented the percentage of invaded bacteria, in comparison to the number of adhered ones, per 10^6^ of cells. *Escherichia coli* K-12 C600 strain was used as the negative control, whereas *Yersinia enterocolitica* O:8/1B was used as a positive control in adhesion and invasion tests.

### Cytotoxic assay

Bacteria were grown overnight in TSB at 37 °C, for 24 h on a rotary shaker. Next, the cultures were centrifuged, at 2000x*g*, for 30 min and sterilised through 0.45 µm PVGF-membrane filters (Roth). Two-fold dilutions (from 1:2 to 1:512) of supernatants were prepared, in phosphate buffered saline (PBS, Biomed). They were added to the HeLa cell monolayer and incubated for 24 h. The effect of extracellular toxins was expressed as a cytotoxic index and assessed as the reciprocal of the highest dilution of the culture filtrates, which produced a cytopathic effect that was observed under an inverted microscope (Krzymińska et al. 2012a, b). As a negative control, cells treated with non-pathogenic *E. coli* K12C600 filtrate were used.

### Statistical analysis

Data on adhesion, invasion and cytotoxic indexes are presented as mean ± standard deviations (SD); they represent two independent experiments, performed in triplicate. A one-way analysis of variance ANOVA with Tukey’s post hoc test, was performed. Linear regression analysis was used to examine the pairwise correlation between adhesion, invasion and cytotoxic indexes, and a Pearson correlation coefficient was determined. p values of < 0.05 were considered statistically significant.

### SCC*mec* typing

Genomic DNA was isolated from isolates using a Genomic DNA Plus kit (A&A Biotechnology, Poland). SCC*mec* types were identified using multiplex PCR (Zhang et al. 2005). Moreover, detection of *ccrAB4* was performed by PCR using primers described by Oliviera et al. (2006). Amplification products were electrophoresed in a 1.5% agarose gel. Gels were stained with ethidium bromide, visualised under a UV light transilluminator, and documented with a V.99 Bio-Print system (Vilber Lourmat, Torcy, France).

### Detection of AME resistance genes

For the detection of resistance genes: *aac(6′)/aph(2″), aph (3′)*-*IIIa* and *ant(4′)*-*Ia,* PCR assays were performed (Ardic et al. 2006).

## Results

### *Staphylococcus hominis* adhesion and invasion of human epithelial cells

All *S. hominis* strains had the ability to adhere to human epithelial cells (Table 1). The adhesion index ranged from 4 × 10^5^ to 5.9 × 10^8^ CFU/ml. The highest index, from 1.4 × 10^8^ to 5.9 × 10^8^ CFU/ml, was evident for four isolates (13%). The lowest index, equal to 4 × 10^5^ CFU/ml, was observed in one strain (1%). The negative control, *E. coli* K12C600 showed an adhesion index of 1.1 × 10^2^ CFU/ml, whereas that of *Y. enterocolitica* O:8/1B (positive control) was 2.6 × 10^7^ CFU/ml. Thirteen *S. hominis* strains (43%) exhibited the ability to invade epithelial cells (Table 1). The invasion indices ranged from 0 to 42%. The highest invasive ability, with an index that ranged from 31 to 42% was observed for five isolates (17%). The index of *Y. enterocolitica* O:8/1B reached 57.3%. The nonpathogenic *E. coli* K12C600 strain did not demonstrate any invasion of epithelial cells.

**Table 1:** Adhesion, invasion and cytotoxic indices of *S. hominis* strains and presence of antibiotic resistance genes as well diversity of the *SCCmec* element

Strain no.	Adhesion index (× 10^7^)	Invasion index (%)	Cytotoxic index (titre)	*MecA* gene	SCC*mec* type			AME resistance genes		
					*Mec* class	*Ccr* type	SCC*mec* type	*Aac(6′)/aph (2″)*	*Aph (3′)*-*IIIa*	*Ant(4* *′)*-*Ia*
MPU Sh13	0.04 ± 0.03^a.^	17.9 ± 8.7^b^	53.3 ± 18.5^c^	+	A	ND	NT	−	+	+
MPU Sh16	0.11 ± 0.07	29.5 ± 21.4	102.1 ± 73.9	+	B	ND	NT	+	+	−
MPU Sh30	0.12 ± 0.06	0	1.3 ± 0.6	+	A	ND	NT	+	−	−
MPU Sh26	0.16 ± 0.12	15.6 ± 9.7	5.3 ± 2.3	+	A	*ccrAB3*	III	+	−	+
MPU Sh27	0.36 ± 0.19	31.4 ± 27.8	85.3 ± 36.9	+	A	ND	NT	−	−	+
MPU Sh28	0.40 ± 0.17	0	37.3 ± 24.4	+	A	*ccrAB4*	VIII	−	+	+
MPU Sh17	0.43 ± 0.25	35.4 ± 9.6	104.7 ± 36.9	+	A	ND	NT	−	−	+
MPU Sh11	1.37 ± 0.74	0	1.7 ± 0.6	+	A	ND	NT	−	−	+
MPU Sh22	1.43 ± 0.80	0	2.7 ± 1.1	+	A	ND	NT	−	+	−
MPU Sh21	1.90 ± 1.25	0	4.7 ± 3.1	+	B	ND	NT	−	−	−
MPU Sh15	2.67 ± 0.74	0	1.7 ± 0.6	+	A	ND	NT	−	+	−
MPU Sh5	3.77 ± 1.68	0	1.3 ± 0.6	+	A	ND	NT	+	+	+
MPU Sh20	4.13 ± 1.93	0	2.3 ± 0.5	+	A	*ccrAB3*	III	−	+	−
MPU Sh29	4.57 ± 1.05	0	1.3 ± 0.6	+	A	ND	NT	−	−	+
MPU Sh14	4.77 ± 1.95	21.6 ± 13.9	37.3 ± 24.4	+	B	ND	NT	+	−	**+**
MPU Sh8	4.95 ± 2.32	0	9.3 ± 6.1	+	A	ND	NT	−	−	+
MPU Sh18	4.97 ± 1.39	37.2 ± 48.2	106.7 ± 36.9	+	A	*ccrAB4*	VIII	−	+	−
MPU Sh12	5.07 ± 2.15	33.8 ± 31.9	74.7 ± 48.9	+	A	ND	NT	−	+	+
MPU Sh23	6.03 ± 1.50	0	3.3 ± 1.4	+	B	ND	NT	+	−	+
MPU Sh25	6.53 ± 0.86	0	2.3 ± 1.5	+	A	ND	NT	−	+	−
MPU Sh19	6.63 ± 2.41	41.7 ± 29.3	105.3 ± 36.9	+	A	ND	NT	−	−	−
MPU Sh10	7.73 ± 1.62	21.3 ± 19.8	37.3 ± 24.4	+	A	*ccrAB3*	III	−	−	+
MPU Sh3	8.53 ± 2.05	0	2.3 ± 1.5	+	A	ND	NT	−	**+**	+
MPU Sh1	9.40 ± 2.98	0	1.3 ± 0.6	+	A	ND	NT	−	−	+
MPU Sh4	9.60 ± 4.88	0	4.0 ± 2.5	+	C	ND	NT	−	+	−
MPU Sh6	9.67 ± 2.80	0	1.7 ± 0.6	+	B	ND	NT	−	+	+
MPU Sh7	14.03 ± 4.16	17.8 ± 11.9	74.7 ± 48.9	+	A	ND	NT	−	−	+
MPU Sh24	14.07 ± 3.20	0	2.3 ± 1.5	+	A	ND	NT	−	−	+
MPU Sh2	57.17 ± 6.88	5.2 ± 3.8	42.7 ± 18.5	+	A	ND	NT	−	−	+
MPU Sh9	58.90 ± 15.15	12.7 ± 10.6	74.7 ± 48.9	+	A	*ccrAB1*	NT	−	+	−

All results are the means ± standard deviation of two separate experiments performed in triplicate

*ND* not detected, *NT* non-typeable SCC*mec* type

^a^Data are expressed as the mean of total number of adherent bacteria per 1 ml of HeLa cells

^b^The percentage of invaded bacteria in comparison with the number adhering

^c^Reciprocal of the highest titre of bacterial culture supernatant that caused destruction monolayer of HeLa cells

### Extracellular cytotoxic activity

All *S. hominis* strains demonstrated activity of extracellular toxins (Table 1). The strains caused a cytopathic effect, which was observed as a rounding off of the HeLa cells. The cytotoxicity titres ranged from 1 to 107. The highest effect, titres ≥ 70, was observed for eight strains (27%), whereas the lowest cytotoxicity, in the range from titre 1 to 5 was observed for 16 isolates (53%). The *E. coli* K-12 C600 strain was not cytotoxic to epithelial cells.

### Analysis of SCCmec structure

All *S. hominis* isolates carried *mecA,* as detected by PCR. Three strains were assigned to SCCmec type III, containing *mec* complex A and *ccrAB3*. Two strains harboured SCC*mec* type VIII i.e. *mec* complex A and *ccrAB4*. The remaining isolates (83%) harboured the non-typeable SCC*mec* type. One isolate carried *ccrAB1* and the class A *mec* complex. For 24 *S. hominis* isolates, no *ccr* gene was identified. A high prevalence (80%) of the *mec* complex A was observed in *S. hominis*. Five strains carried *mec* complex B, whereas one strain carried the *mec* complex C.

### Resistance to aminoglycosides

Of the 30 methicillin-resistant *S. hominis* isolates, 28 (93%) were resistant to aminoglycoside antibiotics. PCR analysis revealed the existence of the *ant(4′)*-*Ia* gene in 19 (63%) strains, *aac(6′)/aph(2″)* in 6 (20%) and *aph(3′)*-*IIIa* in 14 (47%) (Table 1). One strain contained three AME genes. Five isolates (17%) contained two AME genes (*ant(4′)*-*Ia* and *aph(3′)*-*IIIa*), simultaneously. Three *S. hominis* strains carried *aac(6′)/aph(2″)* and *ant(4′)*-*Ia,* whereas only one isolates harboured *aac(6′)/aph(2″)* and *aph(3′)*-*IIIa.* Only two strains did not harbour any AMEs.

## Discussion

Some reports have noted that *S. hominis* is the third most commonly isolated species among CoNS. Nevertheless, the mechanisms of its virulence are not sufficiently understood. Bacterial adherence to epithelia is typically the first, and the essential step in the colonisation of human tissues and the establishment of infections. Bacterial adhesion was studied using only HeLa cells lines, which is a limitation of this work. All *S. hominis* strains have the ability to adhere to HeLa cells. For 18 isolates (60%) the adhesion index was higher than that of the enteroivasive *Y. enterocolitica* O:8/1B positive control strain. The results are consistent with the prevalence of *S. hominis* strains on epithelia. Bacterial adhesion to host cells is mediated by surface-binding proteins and exopolymers (Otto 2010; Becker et al. 2014). The presence of adhesins in *S. hominis* strains was not tested, which is a limitation of this work. Our earlier results revealed that all *S. hominis* strains tested in this study harbour the *ica*ADBC genes encoding the polysaccharide intercellular adhesin (PIA) (Szczuka et al. 2015).

We observed that 43% of the *S. hominis* strains tested had the ability to invade epithelial cells. This could be an important mechanism leading to bacterial evasion of the host’s immune defences and infection maintenance. Some CoNS produce multifunctional adhesin/autolysin (AtlE) that binds fibrinogen, fibronectin and vitronectin in the host, extracellular matrix. Becker et al. (2014) suggested that effect of AtlE could be a possible mechanism for CoNS adhesion and the invasion of host cells.

Bacterial pathogens have evolved remarkable mechanisms to efficiently infect host organisms. In this study, we observed that all *S. hominis* isolates exhibited a cytotoxic effect on human epithelial cells. A statistical analyses demonstrated positive correlations between the invasion and cytotoxic indices (r = 0.79, p < 0.05). It has been demonstrated that *S. epidermidis* strains produce extracellular proteases, metalloproteases, lipases and esterases (Becker et al. 2014). Otto (2012) reported that phenol soluble modulin delta (PSMδ) produced by *S. epidermidis* strains is a potent toxin that exhibits a cytotoxic effect on human cells. These enzymes and toxins induced the destruction of host tissues and the facilitation bacterial invasion.

Another aspect examined in our study was resistance to β-lactams and aminoglycosides i.e. the antibiotics most commonly used to treat staphylococcal infections. These results showed a high prevalence of antibiotic resistance genes, indicating that these strains can be considered as potential reservoirs for these genes in the hospital environment. Importantly, these genes are located on plasmids, transposons and other mobile genetic elements and can be transferred to more pathogenic species. Coexistence of the *mecA* gene and an AME was detected in 93% of the *S. hominis* isolates. The most prevalent AME gene was *ant(4′)*-*I,* which was found in 73% of S*. hominis* isolates. Similar results were obtained in a study by Ida et al. (2001). It was reported that the *ant(4′)*-*I* gene was the most frequent in MRSA strains isolated in Japan. In contrast to our findings and that of Ida et al. (2001). Ardic et al. (2006) demonstrated in a study carried out on MRSA and CoNS, that the prevalence of the *ant(4′)*-*I* gene was 24%, whereas the most frequently encountered AME gene was *aac(6′)/aph (2″).* In this study, *aac(6′)/aph(2″)* was present in only 20% of the strains tested.

This study highlights the high frequency of the *mec* complex A in *S. hominis*. The class B *mec* gene complex was found in only five *S. hominis* strains, whereas only one isolate carried *mec* complex C. Importantly, we did not identify the *ccr* genes in most of the isolates. This failure of identification may be due to the existence of novel allotypes with too low homology with the previously described *ccr* to be detected by the PCR primers used. We can exclude the possibility of the loss of the *ccr* complex from the SCC*mec*. Only five isolates carried a known SCC*mec* type, three of them type III and two SCC*mec* type VIII. Previous studies have also shown that *S. hominis* carries SCC*mec* type VIII and type III (Bouchami et al. 2011; Zhang et al. 2013). The prevalence of non-typeable SCC*mec* observed in this study was previously observed in *S. hominis* (Hanssen and Ericson Sollid 2007; Bouchami et al. 2011; Zhang et al. 2013).

This study provides a new insight into the mechanisms of *S. hominis* pathogenicity. We demonstrated that these strains have the ability to adhere, invade host cells and cause direct epithelial barrier dysfunction. These mechanisms may result in the spreading of bacteria and the development of invasive diseases. Moreover, the bacteria may act as a reservoir for antibiotic resistance genes in the hospital environment.
